# Human antibody repertoire among kidney donors with and without HIV

**DOI:** 10.1172/jci.insight.203645

**Published:** 2026-03-12

**Authors:** Xianming Zhu, William R. Morgenlander, Diane M. Brown, Yolanda Eby, Megan Morsheimer, Jonah Odim, Serena M. Bagnasco, Meenakshi M. Rana, Sander S. Florman, Rachel J. Friedman-Moraco, Peter G. Stock, Alexander J. Gilbert, Shikha Mehta, Valentina Stosor, Sapna A. Mehta, Marcus R. Pereira, Catherine B. Small, Michele I. Morris, Jonathan Hand, Saima Aslam, Ghady Haidar, Maricar Malinis, Carlos A.Q. Santos, Joanna Schaenman, David Wojciechowski, Karthik Ranganna, Emily Blumberg, Nahel Elias, Josa A. Castillo-Lugo, Emmanouil Giorgakis, Senu Apewokin, M. Kate Grabowski, Dorry L. Segev, Andrew D. Redd, Christine M. Durand, H. Benjamin Larman, Aaron A.R. Tobian, on behalf of the HOPE in Action investigators

**Affiliations:** 1 Department of Epidemiology, Bloomberg School of Public Health,; 2 Department of Pathology, School of Medicine, and; 3 Department of Medicine, School of Medicine, The Johns Hopkins University, Baltimore, Maryland, USA.; 4 Division of Allergy, Immunology and Transplantation, National Institute of Allergy and Infectious Diseases, NIH, Bethesda, Maryland, USA.; 5 Department of Medicine, Icahn School of Medicine at Mount Sinai, New York, New York, USA.; 6 Recanati-Miller Transplantation Institute, The Mount Sinai Hospital, New York, New York, USA.; 7 Department of Medicine, Emory University, Atlanta, Georgia, USA.; 8 Department of Surgery, University of California, San Francisco, San Francisco, California, USA.; 9 Department of Medicine, Georgetown University, Washington, DC, USA.; 10 Department of Medicine, University of Alabama at Birmingham, Birmingham, Alabama, USA.; 11 Divisions of Infectious Diseases and Organ Transplantation, Northwestern University Feinberg School of Medicine, Chicago, Illinois, USA.; 12 New York University Langone Transplant Institute, New York, New York, USA.; 13 Department of Medicine, Columbia University Irving Medical Center, New York, New York, USA.; 14 Department of Medicine, Weill Cornell Medicine, New York, New York, USA.; 15 Division of Infectious Diseases, University of Miami Miller School of Medicine, Miami, Florida, USA.; 16 Department of Medicine, Ochsner Health, New Orleans, Louisiana, USA.; 17 Division of Infectious Diseases and Global Public Health, University of California, San Diego, La Jolla, California, USA.; 18 Department of Medicine, University of Pittsburgh, Pittsburgh, Pennsylvania, USA.; 19 Section of Infectious Diseases, Yale School of Medicine, New Haven, Connecticut, USA.; 20 Division of Infectious Diseases, Rush University Medical Center, Chicago, Illinois, USA.; 21 Department of Medicine, David Geffen School of Medicine, University of California, Los Angeles, Los Angeles, California, USA.; 22 Department of Medicine, University of Texas Southwestern Medical Center, Dallas, Texas, USA.; 23 Department of Medicine, Drexel University College of Medicine, Philadelphia, Pennsylvania, USA.; 24 Department of Medicine, Perelman School of Medicine, University of Pennsylvania, Philadelphia, Pennsylvania, USA.; 25 Department of Surgery, Massachusetts General Hospital, Boston, Massachusetts, USA.; 26 Department of Medicine, Methodist Health System Clinical Research Institute, Dallas, Texas, USA.; 27 Department of Surgery, University of Arkansas for Medical Sciences, Little Rock, Arkansas, USA.; 28 Department of Medicine, University of Cincinnati College of Medicine, Cincinnati, Ohio, USA.; 29 Department of Surgery and; 30 Department of Population Health, New York University Grossman School of Medicine, New York, New York, USA.; 31 Division of Intramural Research, National Institute of Allergy and Infectious Diseases, NIH, Baltimore, Maryland, USA.; 32 The HOPE in Action Investigators are detailed in Supplemental Acknowledgments.

**Keywords:** AIDS/HIV, Infectious disease, Public Health, Organ transplantation

## Abstract

**BACKGROUND:**

Transplanting kidneys from donors with HIV to recipients with HIV has become standard clinical practice. However, donors with HIV may have higher prevalence of viral and bacterial infections and autoimmunity that could increase allograft rejection in recipients.

**METHODS:**

We included deceased kidney donors (60 with HIV and 41 without HIV) who participated in a multicenter prospective study of HIV kidney transplantation between April 2018 and September 2021. Using phage immunoprecipitation sequencing, we compared the human antibody repertoire (allergens, autoantibodies, viruses, and bacterial toxins) between donors with and without HIV and evaluated their association with recipient allograft rejection. Moderated *t* tests were used to assess reactivity and a multivariate logistic regression model adjusted for donor sex and kidney donor profile index assessed the association between donor adenovirus reactivity and recipient allograft rejection.

**RESULTS:**

Compared with donors without HIV, donors with HIV had lower BMI and were more likely to be African American. The median number of positive autoantibodies was marginally higher among donors with HIV (499 [IQR, 357, 579]) compared with that of donors without HIV (395 [IQR, 256, 538], *P* = 0.058). Donors with HIV additionally had significantly higher antibody reactivity to Epstein-Barr virus and cytomegalovirus (*q* < 0.05). Among all donors with and without HIV, antibodies against adenovirus were significantly associated with increased rejection among recipients, including after adjusting for false discovery (*q* < 0.05) and also adjusting for demographic factors using multivariable logistic regression (odds ratio = 4.97; 95% CI = 1.89–13.61).

**CONCLUSION:**

The presence of antibodies against adenovirus infection in kidney donors with HIV may be associated with allograft rejection.

**TRIAL REGISTRATION:**

ClinicalTrials.gov NCT03500315.

**FUNDING:**

US NIH.

## Abstract 2

Donor with and without HIV have different antibody repertoire. Donor adenovirus antibodies may be associated with allograft rejection.

## Introduction

Historically, end-stage kidney disease (ESKD) in people with HIV (PWH) was driven by HIV-associated damage to the kidney resulting in HIV-associated nephropathy (HIVAN) (1). With the widespread use of antiretroviral therapy (ART), the incidence of HIVAN has decreased, and PWH who are effectively treated can have similar life expectancy to people without HIV in the US (2). However, the prevalence of ESKD among PWH continues to rise, driven by aging-related comorbidities such as diabetes, chronic immune activation, and ART side effects (3–5). Particularly, the risk for ESKD is disproportionally higher among African Americans with HIV because of their lower access to HIV treatment, higher prevalence of unsuppressed viral load, and the presence of APOL1 risk variants compared with White PWH (6–8).

Kidney transplantation is the best available treatment option for ESKD. In particular, it improves 5-year survival by 79% compared with dialysis among PWH who have ESKD (9). However, PWH have higher rates of rejection compared with people without HIV (10–12). One possible mechanism for increased risk for allograft rejection among PWH is that HIV infection can lead to chronic inflammation and immune dysregulation (13, 14). Other proposed mechanisms include ART nephrotoxicity; presumably, interactions between ART and immune suppressants; and delayed graft function that disproportionally affect PWH (15, 16).

PWH have historically had lower access to transplants than people without HIV due to stigma, logistical obstacles, and policy constraints (17, 18). To improve organ access and reduce waiting time in this population, the HIV Organ Policy Equity (HOPE) Act permitted transplantation of organs from donors with HIV to recipients with HIV (19, 20). Transplantation of kidneys from donors with HIV was shown to be safe and noninferior to transplantation of kidneys from donors without HIV in terms of adverse clinical events, including survival, graft loss, and rejection (21). However, the pathophysiology that differentiates clinical courses among transplant recipients of organs with and without HIV remains incompletely characterized.

Altered immune function in donors with HIV likely affects graft function and propensity for rejection. This may derive from altered exposure/immunity to non-HIV pathogens or from immunomodulatory effects intrinsic to HIV infection. Compared with donors without HIV, donors with HIV are known to have higher prevalence of hepatitis B virus, syphilis, cytomegalovirus, and Kaposi Sarcoma–associated herpesvirus (22, 23). Transplant recipients are also at risk for donor-derived infections, including HIV, cytomegalovirus, Epstein-Barr virus, and hepatitis B and C virus (24, 25). However, exposure to other pathogens and potential consequences for graft outcome have not been evaluated. In addition, it is unknown whether autoantibodies associated with HIV infection in donors affect clinical outcomes among transplant recipients. While kidney transplantation between donors and recipients with HIV is clinically safe, characterizing its biological underpinnings of rejection could help better evaluate the safety of this practice at the molecular level and improve the efficacy of this practice.

Phage immunoprecipitation sequencing (PhIP-seq) utilizes programmable phage display to quantify antibody binding to each of hundreds of thousands of peptide epitopes simultaneously in plasma samples (26). Phage-displayed peptide libraries have been generated that span viruses, toxins, autoantibodies, and allergens and are able to quantify >600,000 antibody peptides in plasma samples (27–29). Advantages of PhIP-seq include its comprehensive and uniform coverage of peptidomes, generation of hundreds of thousands of quantitative data points per sample over a wide dynamic range, peptide-level resolution of antibody binding, high sample throughput, and use of large batch libraries that reduce run-to-run variability (30). Owing to those strengths, PhIP-seq has been utilized to characterize antibodies in different types of studies, including research on HIV, autoimmunity, and transplantation (31–33).

In this study, we evaluated the differences in antibody repertoires between kidney donors with and without HIV and how donor antibody repertoire contributes to posttransplant rejection outcomes among corresponding recipients with HIV.

## Results

### Characteristics of the deceased kidney donors.

The parent HOPE in Action study included 146 deceased kidney donors (64 with HIV and 82 without HIV) (Supplemental Table 1; supplemental material available online with this article; https://doi.org/10.1172/jci.insight.203645DS1). In this analysis, we included 101 donors, of which 60 were HIV positive and 41 were HIV negative. Compared with the 45 donors in the HOPE in Action multicenter national study who did not have an available blood sample for antibody profiling, the 101 donors included in this report showed similar characteristics. Exceptions to this were that the profiled donors had a higher proportion of HIV-positive and cytomegalovirus-seroreactive individuals and a lower proportion of individuals with hypertension. Donors included also had a lower median kidney donor profile index (KDPI) — a score from 0 to 100 that predicts donor kidney function, with lower scores indicating longer estimated survival of the kidney — than donors who were excluded.

In the analytical sample of 101 donors, the median KDPI was 38 (IQR, 28, 53) for donors with HIV and 44 (IQR, 33, 63) for donors without HIV (Table 1). Compared with donors without HIV, donors with HIV were more likely to be African American (40% vs. 15%), have lower median BMI (25 vs. 28), and be antihepatitis B core antibody (HBcAb) positive (20% vs. 2%) and anticytomegalovirus positive (95% vs. 68%). Among donors with HIV, 53% were on ART at the time of donation, and 43% had HIV viral suppression (viral load, <200 copies/mL).

### Characteristics of antibody reactomes.

At the time of kidney donation, PhIP-seq was used to detect donor antibodies binding to 19,137 peptides of allergenic proteins (AllerScan), 259,345 peptides spanning the human proteome (HuScan), 92,171 peptides covering microbial protein toxins (ToxScan), and 106,332 peptides spanning viral proteomes (VirScan). For reactivity to each of 29,216 human proteins, the maximum reactivity to any peptide derived from that protein (ProMax) was used to represent the reactivity to that protein. For reactivity to each of 347 viruses (see the Supporting Data Values files), Viral Aggregate Reactivity Score (VARScore) was used to integrate the intensity and breadth of antibody reactivity to all peptides that span that virus’s proteome.

To ensure benchmark effectiveness of VARScore as a representative of seroreactivity to a virus, we compared the PhIP-seq VARScore for HIV subtype B and cytomegalovirus with the positivity of gold standard testing performed in clinical laboratories. Using the recommended default cutoff of 1 for VARScore, the measure of HIV using PhIP-seq showed 100% sensitivity and 100% specificity, and cytomegalovirus showed 99% sensitivity and 81% specificity (Supplemental Figure 1).

### Donor antibody repertoire by donor HIV status.

**Figure 1:**
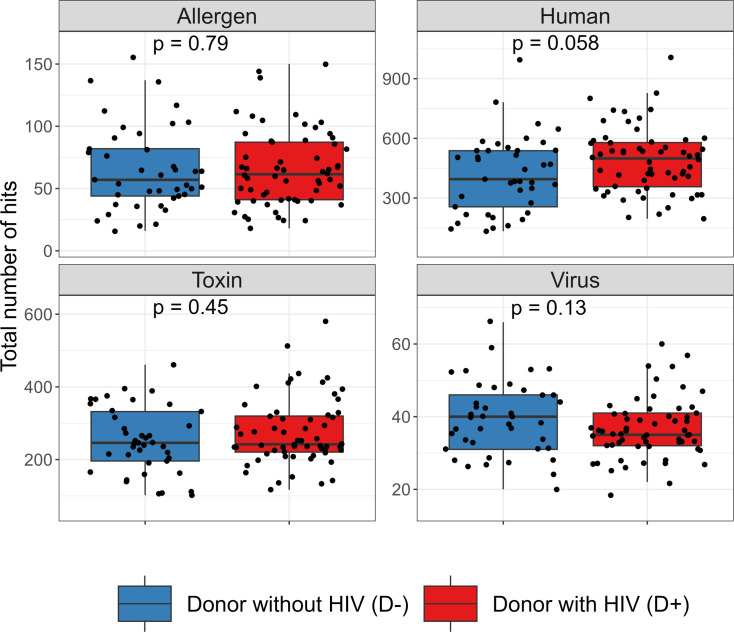
Total number of positive antibody targets among kidney transplant donors with and without HIV. Note: Hit fold change of peptides of allergens and toxins, ProMax of bacterial toxins, or VARScore of viruses >1 was considered positive. Each dot represents the total number of hits of antibody targets for a donor, stratified by their HIV status. HIV viral antibody targets were excluded from the comparison between donors with and without HIV. *P* values were estimated using Wilcoxon’s rank-sum tests.

**Figure 2:**
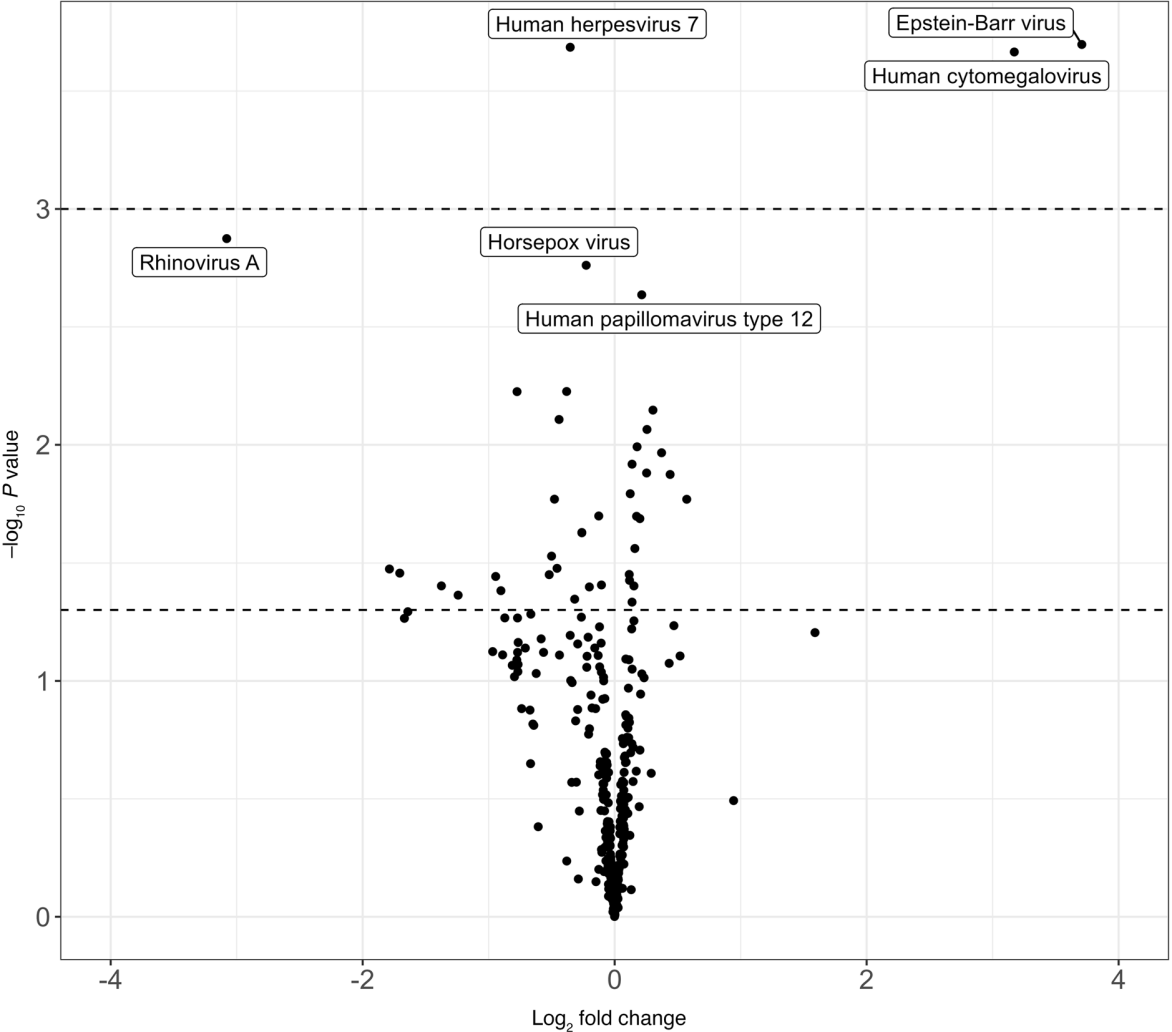
Reactivity of donor viral targets by donor HIV status. Note: Each dot represents the association between donor HIV status and the antibody reactivity to a viral species (measured using continuous VARScore). Moderated *t* tests were used to compute *P* values. Points above the top dashed line represents significant *P* values after correction for multiple comparisons with 5% false discovery rate (*q* < 0.05). Points above the bottom dashed line presents raw *P* < 0.05. Points below the bottom dashed line represents raw *P* ≥ 0.05. Log_2_ fold change >0 suggests donors with HIV had higher reactivity than donors without HIV, while log_2_ fold change <0 suggests donors with HIV had lower reactivity than donors without HIV.

**Figure 3:**
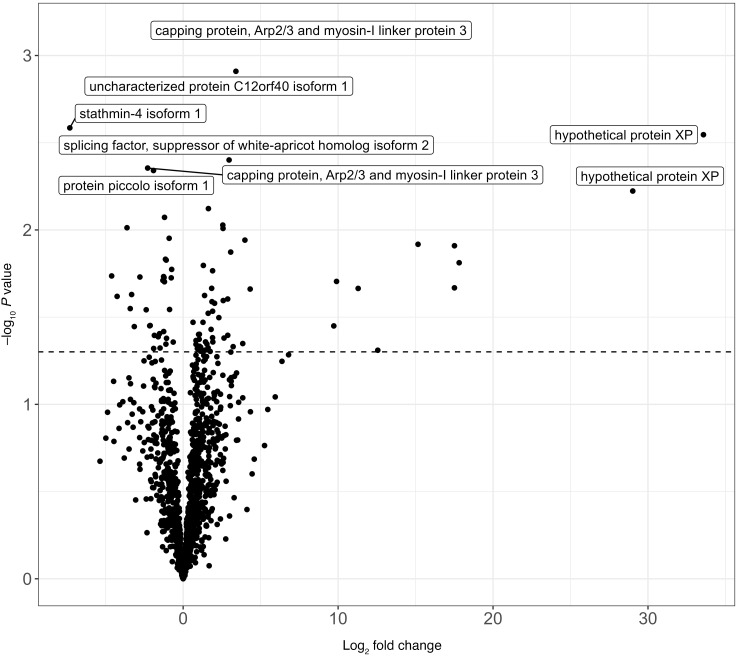
Reactivity of donor human autoantibody targets by donor HIV status. Note: Each dot represents the association between donor HIV status and the antibody reactivity to a peptide of human autoantibody (measured using continuous ProMax). Moderated *t* tests were used to compute *P* values. The dashed line represents *P* = 0.05 (no data points have *q* < 0.05). Log_2_ fold change >0 suggests donors with HIV had higher reactivity than donors without HIV, while log_2_ fold change <0 suggests donors with HIV had lower reactivity than donors without HIV.

The median total number of hits (i.e., positivity) to human peptides among donors with HIV (499 [IQR, 357, 579]) trended higher than that of donors without HIV (395 [IQR, 256, 538]) (*P* = 0.058), while the total number of hits to peptides in toxins and allergy libraries were similar between donors with and without HIV (Figure 1). The overall total reactivity of virus was also similar between donors with HIV and those without HIV, with the exception that donors with HIV had significantly higher reactivity to cytomegalovirus (median [IQR] VARScore, 7.16 [5.33, 9.97] vs. 4.57 [0.46, 6.82]) and Epstein-Barr virus (median [IQR] VARScore, 12.34 [7.82, 15.26] vs. 8.71 [5.32, 11.35]) in addition to having significantly lower reactivity to human herpesvirus 7 than donors without HIV (false discover rate, *q* < 0.05) (Figure 2 and Supplemental Table 2). Measured by PhIP-seq, the positivity was 97% (58/60) vs. 71% (29/41) for cytomegalovirus, 100% (60/60) vs. 100% (41/41) for Epstein-Barr virus, and 2% (1/60) vs. 7% (3/41) for human herpes 7 when comparing donors with HIV with donors without HIV. Donors with HIV trended to have higher reactivity for 52 autoantibodies and lower reactivity for 34 autoantibodies than donors without HIV, but none of them were significant after multiple comparison correction (Figure 3 and Supplemental Table 3).

### Rejection and donor antibody repertoire.

**Figure 4:**
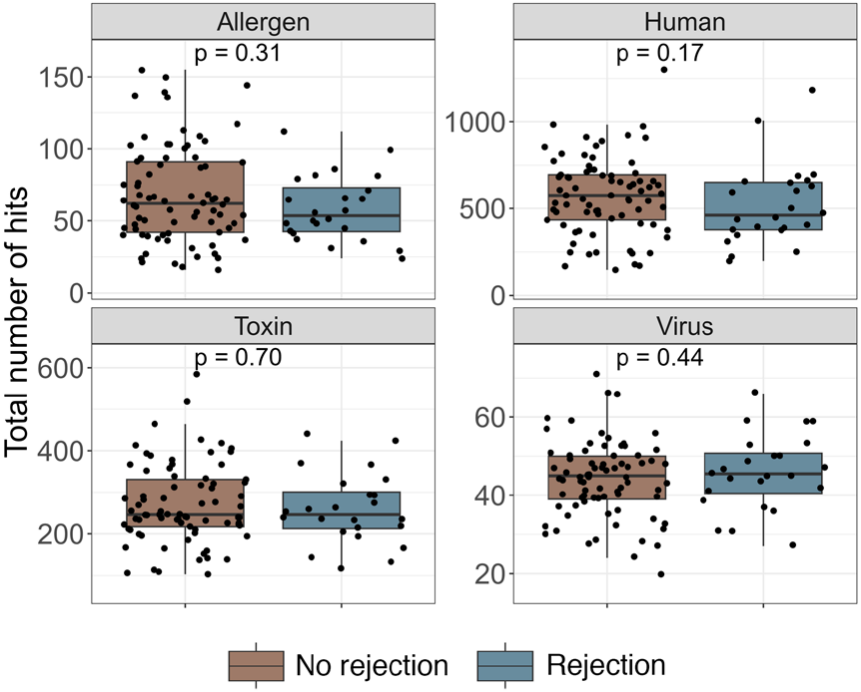
Total number of positive antibody targets among kidney transplant donors by corresponding recipient’s rejection status. Note: Each dot represents the total number of hits of antibody targets for a donor, stratified by the rejection status of their corresponding recipients. Hit fold change of peptides of allergens and toxins, ProMax of bacterial toxins, or VARScore of viruses >1 was considered positive. HIV viral antibody targets were NOT excluded from the comparison. *P* values were estimated using Wilcoxon’s rank-sum tests.

The 101 donors included in this study donated kidneys to 148 recipients. Thirty-five recipients, who received kidneys from 30 donors, experienced allograft rejection after transplant. Both kidneys from 5 donors experienced rejection by a corresponding 10 recipients. Among the 35 rejection episodes, 20 experienced T cell–mediated rejection, 4 had both T cell–mediated and antibody-mediated rejection, and 1 experienced only antibody-mediated rejection. Rejection mechanisms for 10 episodes were uncertain. The median total number of hits was similar for all libraries between donors whose recipients experienced rejection and donors whose recipients did not (Figure 4).

**Figure 5:**
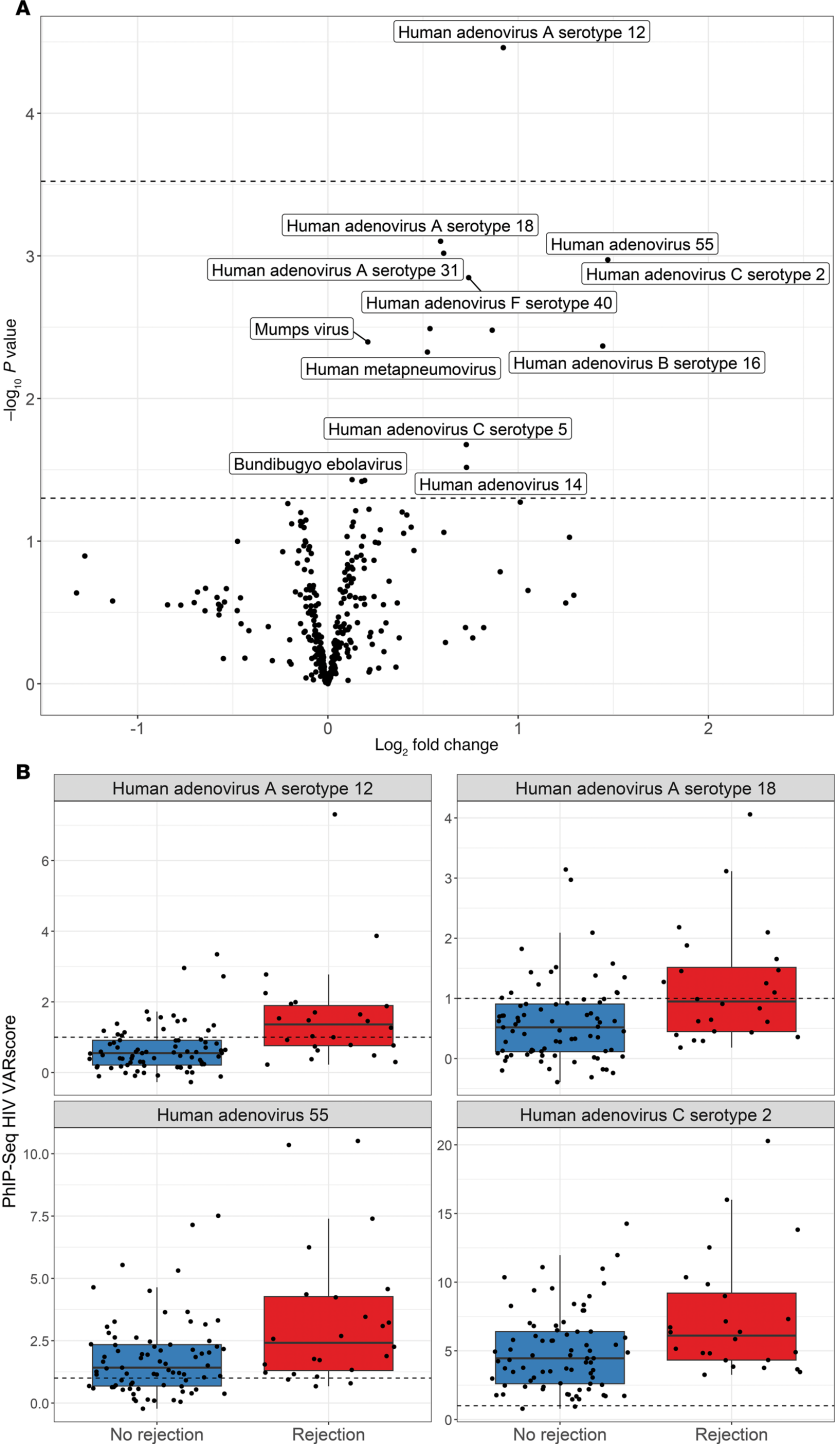
Reactivity to donor viral antibody targets and allograft rejection. (**A**) Note: Each dot represents the association between donor antibody reactivity to a viral species (measured using continuous VARScore) and allograft rejection. Moderated *t* tests were used to compute *P* values. Points above the top dashed line represent significant *P* values after correction for multiple comparisons with 5% false discovery rate (*q* < 0.05). Points above the bottom dashed line present raw *P* < 0.05. Points below the bottom dashed line represents raw *P* ≥ 0.05. Log_2_ fold change >0 represents viral antibody reactivity that was positively associated with rejection, while log_2_ fold change <0 represents viral antibody reactivity that was negatively associated with rejection. (**B**) Note: Each dot represents a donor. Adenovirus species with the 4 smallest *P* values in **A** were selected for the comparison in **B**. VARScore above the horizontal dashed line (>1) represents positivity of antibody to adenovirus.

However, higher reactivity to donor adenovirus A12 (continuous) was significantly associated with recipient allograft rejection (*q* < 0.05), and donor reactivity to other adenovirus types (e.g., A18, 55, and C2) also trended toward positive associations with rejection (*P* < 0.05) (Figure 5A and Supplemental Table 4). Kidneys from 45% (14/31) of donors with presence of antibodies against adenovirus A12 (VARScore >1, binary) experienced rejection, comparing to only 14% (10/70) of donors without such positivity (χ^2^, *P* < 0.001) (Figure 5B). Similar trends were observed among adenovirus subtypes A18, 55, and C2. In a multivariable logistic regression adjusting for sex and KDPI, the presence of donors’ antibodies against adenovirus A12 (binary) was associated with 4.97 (95% CI, 1.89, 13.61) times increased odds of rejection.

**Figure 6:**
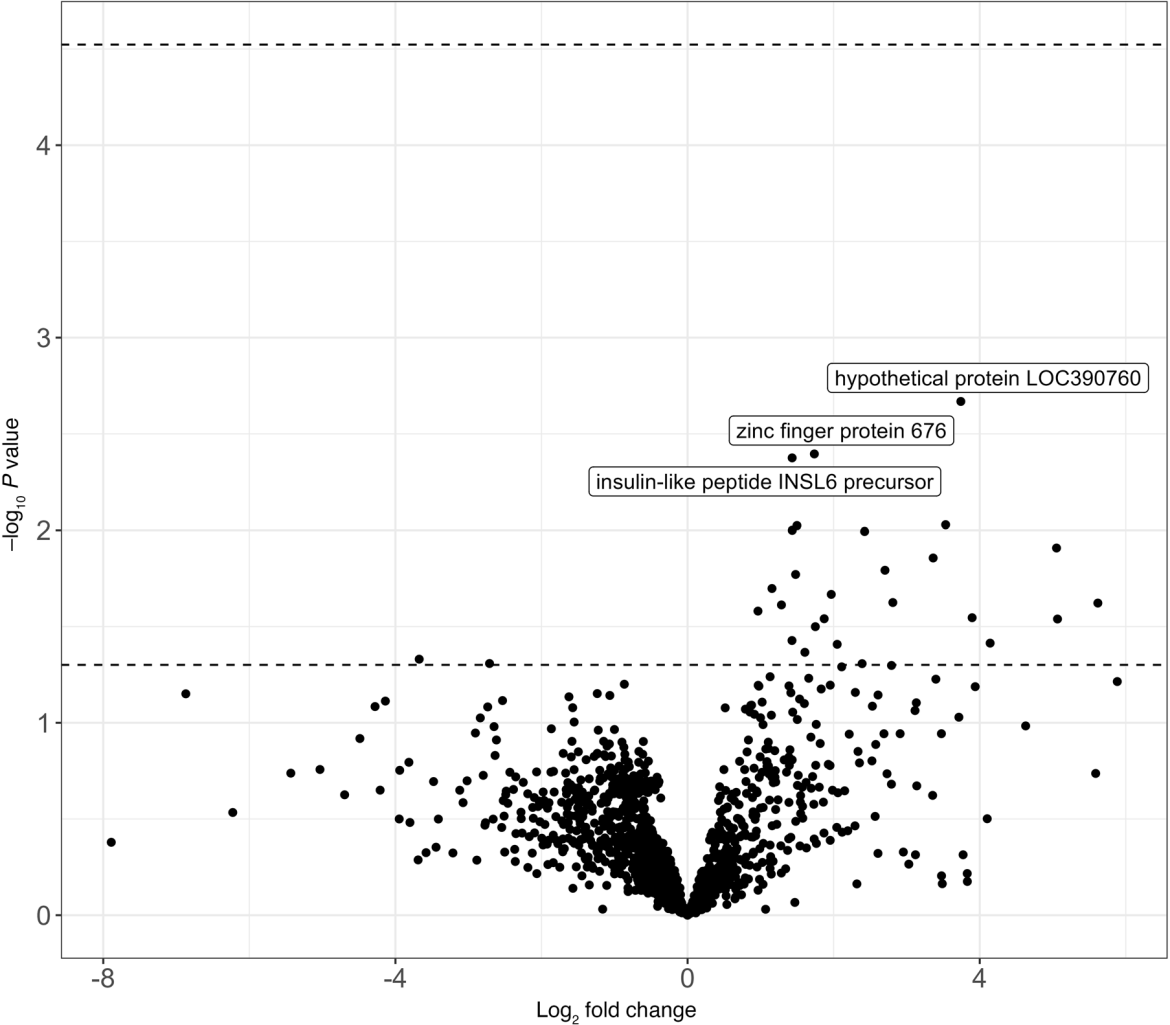
Reactivity of donor human autoantibody targets and rejections among recipients. Note: Each dot represents the association between donor antibody reactivity to a peptide of human autoantibody (measured using continuous ProMax) and allograft rejection. Moderated *t* tests were used to compute *P* values. Points above the top dashed line represent significant *P* values after correction for multiple comparisons with 5% false discovery rate (*q* < 0.05). Points above the bottom dashed line presents raw *P* < 0.05. Points below the bottom dashed line represents raw *P* ≥ 0.05. Log_2_ fold change >0 represents autoantibody reactivity that was positively associated with rejection, while log_2_ fold change <0 represents autoantibody reactivity that was negatively associated with rejection.

Twenty-six autoantibodies were positively associated with recipient rejection and two were negatively associated; however, none remained statistically significant after adjustment for multiple comparisons (Figure 6 and Supplemental Table 5). No clear association with rejection was observed among bacterial toxins and allergens.

## Discussion

In this study, we identified differences in antibody repertoire reactivity profiles between deceased kidney donors with and without HIV using PhIP-seq. Specifically, donors with HIV had higher antibody reactivity to Epstein-Barr virus and cytomegalovirus compared with donors without HIV. We also identified that increased donor antibody reactivity to adenovirus from kidney donors before transplant was significantly associated with higher risk for rejection among corresponding recipients.

We believe that our study is among the first to identify the association between donor adenovirus status and kidney rejection among recipients. While the majority of rejection episodes in our study were cellular mediated, one possible mechanism is that the presence of donor adenovirus antibodies may lead to immune activation or increased alloimmune risk, which could result in allograft dysfunction in recipients (34). Previous studies have mostly focused on recipient adenovirus infection and allograft dysfunction. Adenovirus disease among recipients after transplantation is generally caused by community acquisition, viral reactivation, and donor transmission (24, 35–37). While adenovirus infection usually only causes mild respiratory disease among immunocompetent people, it can cause disseminated disease or nephritis among kidney transplant recipients (38). The effect of adenovirus on risk of kidney rejection is uncertain, and studies on donor-derived adenovirus infection are limited. A recent study with 17 kidney recipients with adenovirus infection showed that 53% developed rejection after infection (39). However, another study among pediatric kidney transplant recipients did not find an association between adenovirus infection and allograft dysfunction (40). Our findings should be interpreted with caution, as IgG reactivity cannot differentiate active from past infections. Additionally, Phip-seq is subject to cross-reactivity among homologous epitopes, particularly within the same viral species, limiting capacity to differentiate exposure to adenovirus subtypes (41). Since adenoviruses are common and their effect on transplantation outcomes are uncertain, they are not routinely screened among kidney donors (42, 43). As PhIP-seq is relatively inexpensive for the coverage obtained and only requires a small amount of blood sample, antibody reactome technologies may also have the potential to serve as an additional tool to screen high risk donors for HIV organ transplantation, including those with the presence of adenovirus antibodies. Our findings will need to be validated in this and other cohorts, preferably with a gold standard assay technology such as ELISA. If validated, specific adenovirus antibodies (or potentially the presence of specific adenovirus) may represent a potential screening biomarker for donors in kidney transplantation to recipients with (and potentially without) HIV.

Donor autoantibodies may affect the outcomes in kidney transplantation among PWH. While organ donors with HIV were generally physically healthier than donors without HIV (lower BMI and KDPI) in our sample and had similar or even lower inflammatory profiles in our previous study (44), donors with HIV trended toward a higher frequency of autoantibody positivity. This finding is consistent with the literature, which shows that HIV infection is associated with increased prevalence of autoantibodies (45). While autoantibodies against human leukocyte antigen (HLA) are known to cause allograft dysfunction, there are fewer data on donor non-HLA autoantibodies (46). While we identified dozens of donor non-HLA autoantibodies that tend to be associated with kidney rejection, owing to the relatively small sample size of this study, we were unable to make inferences on those autoantibodies. However, as a hypothesis-generating study, the findings can help guide future research.

The study is subject to several limitations. First, owing to the small sample size, our findings did not suggest causal role of donor antibodies, and we have limited power to detect peptides/species that have small effect size or evaluate synergistic effect between analytes. However, as an exploratory analysis to formulate hypotheses, our study could guide future causal and intervention studies to validate our findings, including those that fail the cutoff for accounting for multiple comparisons. Second, most rejection episodes were T cell mediated. Because only 1 participant experienced antibody-mediated rejection and 4 experienced both antibody- and T cell–mediated rejection, we were unable to evaluate the relationship between donor antibody profiles and antibody-mediated rejection. However, as the number of transplants between donors and recipients with HIV increases, more cases of antibody-mediated rejection may occur, allowing for a better understanding of the underlying immune mechanisms. Third, PhIP-seq results may be subject to cross-reactivity among antibodies that recognize structurally homologous similar antigens, such as different subtypes within the same viral species. Thus, we were unable to assess the synergistic effect between subtypes. Another limitation is that PhIP-seq uses peptides to represent autoantigens, which lack conformational epitopes and posttranslational modifications. Finally, this study was limited to the donor repertoire in kidney transplantation among PWH. Future studies are needed to assess the effect of the recipient antibody repertoire on transplant outcomes and to replicate these analyses to the general population without HIV.

Using PhIP-seq, we comprehensively evaluated the donor antibody repertoire by HIV status and rejection status in the setting of kidney transplantation to recipients with HIV. Our findings suggest that history of adenovirus infection and autoantibody profiles may represent additional targets considerations for donor screening in HIV-associated kidney transplantation.

## Methods

### Sex as a biological variable.

Sex was treated as a biological variable, and both male and female individuals were included in this study.

### Study population.

HOPE in Action was a multicenter prospective study to evaluate the safety of kidney transplantation from deceased donors with and without HIV to recipients with HIV at 26 centers in the United States from April 2018 to September 2021 (ClinicalTrials.gov NCT03500315) (21). Kidneys were allocated to recipients from organs with or without HIV based on first availability. Donors were screened for HIV using antibody tests and nucleic acid tests performed by the organ procurement organizations (21, 47). Western blot or fourth generation HIV antibody/antigen and quantitative HIV viral load were used for confirmatory testing. False positive donors (i.e., those with a positive screening test but a negative confirmatory test) were considered as donors without HIV (48). Donors with HIV with active opportunistic infections or cancer were not eligible, while there was no restriction on donor HIV viral load or CD4^+^ T cell count. A total of 198 recipients with HIV (99 received organs with HIV and 99 received organs without HIV) agreed to participate and received a kidney transplant from 146 donors (64 with HIV and 82 without HIV).

We collected blood samples from all donors with HIV in the parent HOPE in Action study, and all donors with false positive HIV screening tests, later confirmed as donors without HIV, for a total of 105 donor blood samples from 105 donors (64 with HIV and 41 without HIV) at the time of kidney donation. Four donors with HIV were excluded from this analysis due to missed collection or insufficient sample volume. As a result, 101 donors (60 with HIV and 41 without HIV) were included in the analytical sample of this study. We collected demographic (classifications defined by investigators), clinical, and biological information from donors, including age, biological sex, race, donation after cardiac/brain death, cause of death, steroid administered, KDPI, BMI, hypertension, diabetes, serum creatine, HCV, HBV, and cytomegalovirus status. Information on ART, HIV viral suppression, and CD4^+^ T cell count were also collected from donors with HIV.

Recipient rejection episodes and rejection type (i.e., cellular mediated or antibody mediated) were verified through biopsy and adjudicated by central pathologist review. Donors who had 1 or both donated kidneys rejected were categorized as having a rejection status of “Yes.” Due to the low number of antibody-mediated rejection episodes, our analysis did not stratify by rejection type.

### Sample collection.

Whole blood was drawn from deceased donors and was shipped to the HOPE in Action core laboratory at Johns Hopkins University overnight. Upon arrival, blood samples were centrifuged at 1,000*g* for 5 minutes (with no break) to isolate plasma from red blood cells and were subsequently frozen at −80°C for later processing.

### PhIP-seq.

PhIP-seq was conducted as previously described (26, 49). Briefly, after thawing, we mixed 0.2 μL plasma with libraries of phage-displayed peptides derived from human disease-causing viruses, bacterial toxins, allergens, and the human proteome (27). On each 96-well plate, 6–8 mock controls without plasma were included. Sample antibodies and antibody-bound phage were immunocaptured using protein A– (Thermo Fisher, 10002D) and protein G–coated (Thermo Fisher, 10004D) magnetic beads on 96-well plates. A bravo liquid handling robot was used for bead washing. We resuspended magnetic beads in PCR master mix for thermocycling, and then a second PCR was implemented for sample barcoding. Finally, we pooled the PCR products and sequenced them using Illumina NextSeq 500 to determine the peptides bound to phages. The study included analysis of antibody reactivity to 4 peptide libraries: the human proteome library, the virus library, the toxin library, and the allergen library.

### Statistics.

Sequencing data were processed as previously described (50–52). Through exact sequence matching, we aligned sequencing reads to the peptide library. EdgeR package was used to compare each sample against the set of mock immunoprecipitations to determine the fold over background value and the statistical significance of enrichment. Fold changes comparing sample read counts to negative controls were estimated using exact test for negative binomial distribution. Peptides were classified as hits for a sample if they met the following criteria: a count of at least 100, a *P* value of less than or equal to 0.001, and a fold change of 5 or greater. The hit fold change values indicate the fold changes of these hits, while nonhits are assigned a value of 1.

Antibody reactivity was reported using different metrics depending on whether the reactivity was to an allergen, toxin, virus, or human protein. For reactivity to toxins and allergens, we report reactivity at the peptide level as fold change reactivity compared with negative control. For autoreactivity to human proteins, we report reactivity at the protein level as the maximum reactivity of all peptides tiling a given protein (ProMax). For antibody reactivity to viruses, we report reactivity at the virus level using VARscore to integrate the intensity and breadth of response to all antigen peptides of a virus (ARscore package). We defined reactivity as continuous and positivity of an analyte as the hit fold change, ProMax, or VARScore greater than 1. We performed quality control by evaluating the sensitivity and specificity of the VARScore for HIV subtype B and cytomegalovirus against gold standard testing.

Characteristics of donors excluded and included in this analysis were compared using Wilcoxon’s rank-sum tests for continuous variables and Fisher’s exact tests for categorical variables. Characteristics of 101 donors included in this study were summarized by HIV status. Wilcoxon’s rank-sum tests were used to compare the total number of hits (i.e., positivity) for each library by donor HIV status and by corresponding recipient allograft rejection status. HIV targets were excluded from the comparison of VARscore by HIV status. We used moderated *t* tests to evaluate the reactivity (continuous) of each analyte by HIV and rejection, and analytes with <5% or >95% positivity were excluded. All tests were 2 tailed, and a false discovery rate *q* < 0.05 was considered statistically significant to account for multiple comparisons. We conducted a post hoc evaluation of the association between donor adenovirus reactivity and rejection, using a multivariate logistic regression model adjusted for donor sex and KDPI. All analyses were performed in R version 4.5 (R-Core Team).

### Study approval.

The recipient HOPE study was reviewed and approved by the Johns Hopkins University School of Medicine Institutional Review Board (JHUIRB, IRB00141138) centrally and by each local transplant center (see Transplant centers in the supplemental materials). The donor sample collection and this specific PhIP-seq study were also approved by the JHUIRB (IRB00041681 and IRB00387066, respectively). Written informed consent was provided by all participants.

### Data availability.

Because all data were collected from humans, they are not publicly available. However, deidentified data can be provided upon reasonable request. Proposals to access deidentified data from the HOPE in Action study can be submitted to the steering committee (contact CMD; christinedurand@jhmi.edu), with transfer approved on an individual basis via a formal data use agreement. Detailed data points shown in this manuscript are included in the Supporting Data Values file.

## Author contributions

Conception and design: DLS, ADR, CMD, and AART. Obtaining funding: ADR, DLS, CMD, and AART. Collection and assembly of data: HBL, WRM, DMB, and YE. Provision and clinical care of study participants: MMR, SSF, RJFM, PGS, AJG, SM, VS, SAM, MRP, CBS, OA, MIM, JH, S Aslam, GH, M Malinis, CAQS, JS, DW, KR, EB, NE, JACL, EG, S Apewokin, DLS, and CMD. Conduct and oversight of laboratory operations: HBL, WRM, YE, and AART. Administrative, technical, or logistic support: DMB, YE, M Morsheimer, SMB, and JO. Interpretation of data: XZ, HBL, WRM, MKG, CMD, and AART. Statistical analysis: XZ, HBL, and WRM. Drafting of the manuscript: XZ, CMD, and AART. Critical revision of manuscript for important intellectual content: XZ, HBL, WRM, CMD, and AART. Final approval of the article: All authors.

## Conflict of interest

JH reports receiving research grants from Pfizer, Janssen, Ferring, Scynexis, AstraZeneca, and the Antibiotic Resistance Leadership Group (ARLG). being an advisory board member at Pfizer, AstraZeneca, and Innoviva; and receiving honoraria from the ARLG. DLS reports consulting with AstraZeneca, CareDx, Moderna Therapeutics, Novavax, Regeneron, Springer Publishing, Hansa, Optum, OrgonOx, Medscape, and Roche and receiving honoraria from AstraZeneca, CareDx, Houston Methodist, Sanofi, Northwell Health, Optum Health Education, WebMd, ASN, and Roche. CMD reports receiving payments from Gilead Sciences for serving on a grant review committee, and Gilead donates drugs to two trials on which CMD is an investigator. HBL is an inventor on an issued patent (US 17/583,947) covering the VirScan technology and is a founder of Infinity Bio Inc.

## Funding support

This work is the result of NIH funding, in whole or in part, and is subject to the NIH Public Access Policy. Through acceptance of this federal funding, the NIH has been given a right to make the work publicly available in PubMed Central.

- NIH grants (R01AI20938 and R01DK131926 to AART and U01AI138897 and U01AI177211 to CMD and DLS).
- Intramural Research Program of the NIH (to ADR).

## Supplementary Material

Supplemental data

ICMJE disclosure forms

Supporting data values

## Figures and Tables

**Table 1:**
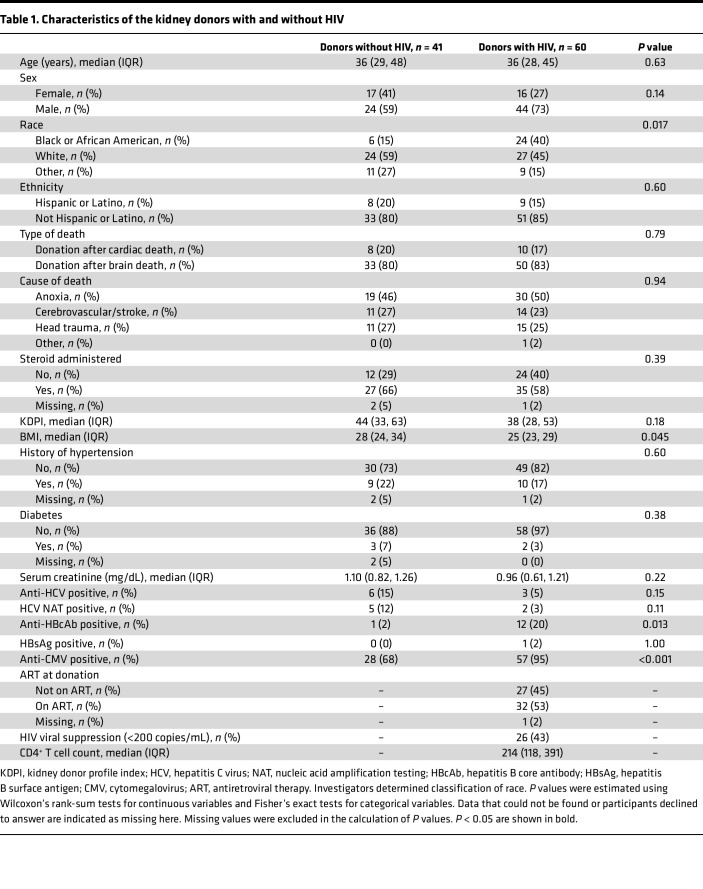
Characteristics of the kidney donors with and without HIV

## Version 1 03/12/2026

In-Press Preview

## Version 2 04/22/2026

Electronic publication
