# Supplementary material for: Human antibody repertoire among kidney donors with and without HIV
Source: JCI Insight. 2026 Mar 12;11(8):e203645. doi: 10.1172/jci.insight.203645 (PMC13135408; doi:10.1172/jci.insight.203645)
Supplement: Supplemental data [file jciinsight-11-203645-s221.pdf]

Acknowledgements

Hope in Action Investigator Teams

Affiliation	Name
Columbia University Medical Center	Brittney Destin; Marcus Pereira; Theresa Lukose; Dominque Piquant;
David Geffen School of Medicine UCLA	Suphamai Bunnapradist; H. Albin Gritsch; Rosemary Silva; Adreanne Rivera; Jeffrey Veale
Drexel University	Dong Heun Lee; Karthik Ranganna
Emory University Transplant Center	Rivka Elbein; Elizabeth Ferry; Jeryl Huckaby; William Kitchens; G. Marshall Lyon; Aneesh K. Mehta; Thomas Pearson; April Roberson
Indiana University	Oluwafisayo Adebiyi; Margaret Adebiyi
Johns Hopkins University	Olivia M. Akinde; Serena Bagnasco; Brittany Barnaba; Gilad Bismut; Mary Grace Bowring; Diane Brown; Maggie Chahoud; Willa Cochran; Berlie DeJen; Niraj Desai; Christine Durand; Yolanda Eby; Reinaldo Fernandez; Naqvi Fizza; Feben Habtehyimer; Sarah Hussain; Morgan Keruly; Charles Kirby; Tao Liang; Jernelle Miller; Darin Ostrander; Michelle Prizzi; Grace Rozek; Jessica Ruff; Isabella Sengsouk; Haley Schmidt; Aaron Tobian; James Wiles; William Werbel; Xianming Zhu
Massachusetts General Hospital	Nahel Elias; Olivia Hess; Margaret Thomas; Kerry Crisalli
MedStar Georgetown Transplant Institute	Alexander Gilbert
Methodist Health System Clinical Research Institute	Karen Castro
Mount Sinai Hospital, Recanati-Miller Transplantation Institute	Sander S. Florman; Brandy Haydel; Shirish Huprikar; Meenakshi M. Rana
National Institute of Allergy and Infectious Diseases, National Institutes of Health	Erica Brittain; Megan Morsheimer; Jonah Odim; Thomas Quinn; Andrew Redd; Natasha Watson
New York University Langone Transplant Institute	Rebecca Dieter; Allan Massie; Sapna Mehta; Jennifer D. Motter; Henry Neumann; Dorry Segev
Northwestern University	Michelle Callegari; Leah Goudy; Valentina Stosor
Ochsner Health	Jonathan Hand; Angela Smith; Ari Cohen
Perelman School of Medicine at the University of Pennsylvania	Emily Blumberg; Maryann Najdzinowicz
Positive Rhetoric LLC, Bowling Green, KY	Brianna Doby
Rush University Medical Center	Mark Mall; Carlos A. Santos
University of Alabama at Birmingham	Katherine Basinger; Jayme Locke; Shikha Mehta; Darnell Mompoint-Williams

University of Arkansas for Medical Sciences	Sushma Bhusal; Emmanouil Giorgakis; Rebecca Wilson
University of California, San Diego	Saima Aslam; Kristin Mekeel; Layla Myers; Mita Shah
University of California, San Francisco	Ada Chao; Monica Fung; Peter Chin Hong; Garrett Roll; Rodney Rogers; Peter Stock
University of California, Los Angeles	Joanna Schaenman
University of Cincinnati	Senu Apewokin; Madison Cuffy; Samantha Kramer; Shimul Shah; Racheal Wilkinson
University of Maryland, Institute of Human Virology	Lisa Anderson; John Baddley
University of Miami, Miami Transplant Institute	Shweta Anjan; Adela Mattiazzi; Lissett Moni; Michele Morris; Carlos Munoz Jacques Simkins; Isabel Vital
University of Pittsburgh Medical Center	Ghady Haidar; Ken Ho; Kailey Hughes Kramer; Sarah McBeth; Diana L. Pakstis; Fernanda Silveira
University of Texas Southwestern Medical Center	Ricardo M. La Hoz; Jarrett Hubbard; Swee-Ling L. Levea; Jennifer Nixon; Parsia Vagefi; David Wojciechowski
Weill Cornell Medicine	Candace Alleyne; Anna Gwak; Thangamani Muthukumar; Catherine Small; Britta Witting
Yale School of Medicine	Richard Formica; Sanjay Kulkarni; Maricar Malinis; Ricarda Tomlin

Transplant centers

**HOPE in Action transplant center**	**Location**
Mount Sinai Medical Center (NYMS)	New York City, NY
Johns Hopkins Hospital (MDJH)	Baltimore, MD
Emory University Hospital (GAEM)	Atlanta, GA
MedStar Georgetown Transplant Institute (DCGU)	Washington DC
University of California San Francisco Medical Center (CASF)	San Francisco, CA
NYU Langone Health (NYUC)	New York City, NY
University of Alabama Hospital (ALUA)	Birmingham, AL
Northwestern Memorial Hospital (ILNM)	Chicago, IL
NY Presbyterian/Columbia University Medical Center (NYCP)	New York City, NY
University of Miami School of Medicine (FLJM)	Miami, FL
Ochsner Foundation Hospital (LAOF)	New Orleans, LA
University of California San Diego Medical Center (CASD)	San Diego, CA
University of Pittsburgh Medical Center (PAPT)	Pittsburgh, PA
Yale New Haven Hospital (CTYN)	New Haven, CT
NY Presbyterian Hospital/Weill Cornell Medical Center (NYNY)	New York City, NY
Rush University Medical Center (ILPL)	Chicago, IL
University of California at Los Angeles Medical Center (CAUC)	Los Angeles, CA
University of Maryland Medical Center (MDUM)	Baltimore, MD

UT Southwestern Medical Center (TXSP)	Dallas, TX
Hahnemann University Hospital (PAHM)*	Philadelphia, PA
Hospital of the University of Pennsylvania (PAUP)	Philadelphia, PA
Indiana University Health (INIM)	Indianapolis, IN
Massachusetts General Hospital (MAMG)	Boston, MA
Methodist Dallas Medical Center (TXMC)	Dallas, TX
University of Arkansas Medical Center (ARUA)	Little Rock, AR
University of Cincinnati Medical Center (OHUC)	Cincinnati, OH

* Two participants from PAHM were transferred to PAUP, since PAHM were closed during the study.

Supplemental Table 1. Characteristics of the kidney donors with PhIP testing vs without

	Donor included<br>n = 101	Donor excluded<br>n =45	p value
Donor with HIV	60 (59)	4 (9)	**<0.001**
Age, years, median (IQR)	36 (29, 47)	43 (32, 49)	0.12
Female sex, n (%)	33 (33)	11 (24)	0.34
Race, n (%)			0.81
Black or African American	30 (30)	13 (29)	
White	51 (50)	25 (56)	
Other	20 (20)	7 (15)	
Ethnicity			1.00
Hispanic or Latino	17 (17)	7 (16)	
Not Hispanic or Latino	84 (83)	38 (84)	
Type of death, n (%)			0.38
Donation after cardiac death, n (%)	18 (18)	11 (24)	
Donation after brain death, n (%)	83 (82)	34 (76)	
Cause of death, n (%)			0.057
Anoxia	50 (50)	17 (38)	
Cerebrovascular/Stroke	25 (25)	14 (31)	
Head Trauma	26 (26)	11 (24)	
Other	0 (0)	3 (7)	
Steroid administered, n (%)			0.70
No	36 (36)	14 (31)	
Yes	62 (61)	30 (67)	
Missing	3 (3)	1 (2)	
KDPI, median (IQR)	40 (28, 58)	55 (35, 70)	**0.024**
Body mass index, median (IQR)	26 (23, 30)	28 (23, 33)	0.43
History of hypertension, n (%)			**0.023**
No	79 (78)	28 (62)	
Yes	19 (19)	17 (38)	
Missing	3 (3)	0 (0)	
Diabetes, n (%)			0.30
No	94 (93)	40 (89)	
Yes	5 (5)	5 (11)	
Missing	2 (2)	0 (0)	
Serum creatinine, mg/dL, median (IQR)	1.01 (0.75, 1.21)	1.00 (0.80, 1.41)	0.38
Anti-HCV positive, n (%)	9 (9)	4 (9)	1.00
HCV NAT positive, n (%)	7 (7)	3 (7)	1.00
Anti HBcAb positive, n (%)	13 (13)	2 (4)	0.15
HBsAg positive, n (%)	1 (1)	1 (2)	1.00
Anti CMV positive, n (%)	85 (84)	30 (67)	**0.027**

Abbreviation: KDPI: Kidney Donor Profile Index; HCV: hepatitis C virus; NAT: Nucleic Acid Amplification Testing; HBcAb: hepatitis B core antibody; HBsAg: Hepatitis B surface antigen; CMV: cytomegalovirus.

P values were estimated using Wilcoxon rank sum tests for continuous variables and fisher's exact tests for categorical variables.

Missing values were excluded in the calculation of p values.

P<0.05 were in bold.

Supplemental Figure 1. Comparison of measured antibody reactivity using PhIP-Seq with measurement using gold standard testing.

Note: Abbreviation: CMV: cytomegalovirus.
Each dot represents a donor. VARScore above the horizontal dashed line (>1) represent positivity of antibody to CMV or HIV.

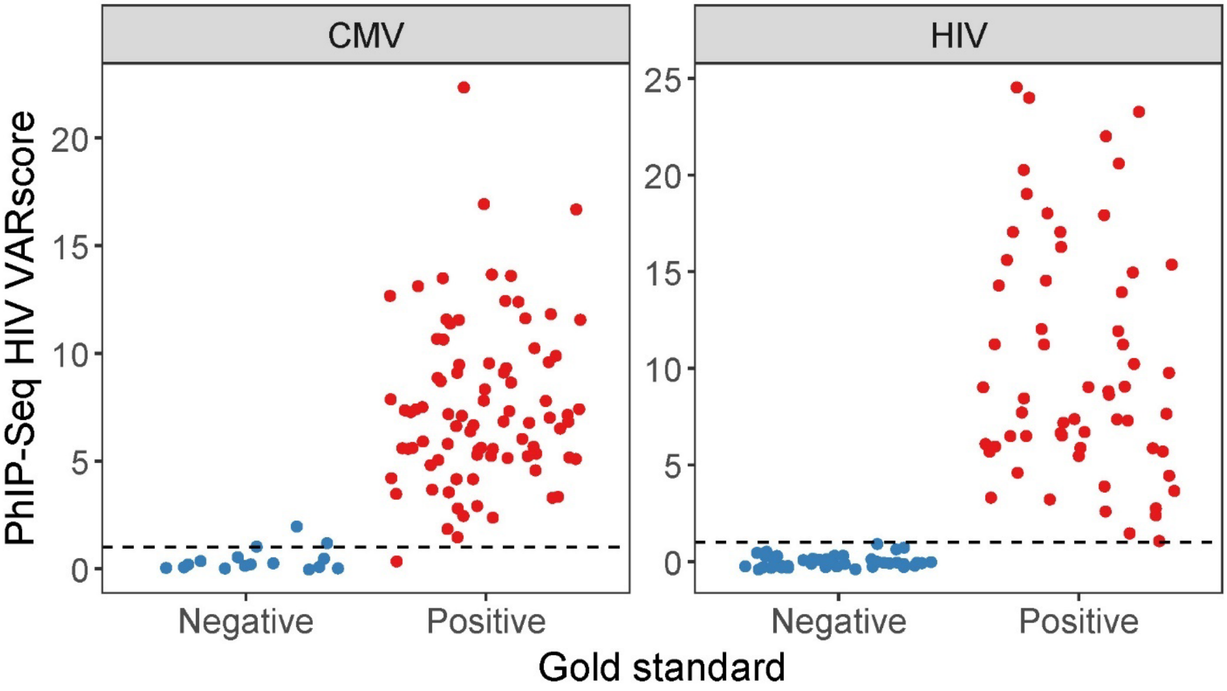

Supplemental Table 2. Reactivity of donor viral targets by donor HIV status (extension of Figure 2)

Note: Only viral species with raw p value<0.05 were included in this table. Log2 fold change represents the log2 ratio of a viral species between donors with HIV vs donors without HIV, and a positive log2 fold change suggests reactivity of the viral species was positively associated with donor HIV status.

log2 fold change	p value	q value	Viral species
3.70767582	0.0002007	0.02411442	Epstein-Barr virus
-0.352635	0.00020621	0.02411442	Human herpesvirus 7
3.17180971	0.00021595	0.02411442	Human cytomegalovirus
-3.0792569	0.00133573	0.11186744	Rhinovirus A
-0.2261181	0.00173285	0.11610096	Horsepox virus
0.21482329	0.00231181	0.12907581	Human papillomavirus type 12
-0.3811709	0.00594159	0.24923098	Human astrovirus-6
-0.7747952	0.00595178	0.24923098	Human herpesvirus 6B
0.30383052	0.00712755	0.26164088	Canine kobuvirus US-PC0082
-0.4412425	0.00781018	0.26164088	Human papillomavirus type 37
0.25644795	0.00861007	0.26221579	Crimean-Congo hemorrhagic fever virus
0.17784953	0.01019752	0.27857057	Dhori virus
0.37228543	0.0108102	0.27857057	Human adenovirus D serotype 17
0.1380726	0.01208327	0.27975611	Hepatitis E virus
0.25348765	0.01317035	0.27975611	Rosavirus M-7
0.44025043	0.01336149	0.27975611	Human herpesvirus 8 type P
0.12359219	0.01612548	0.30011225	Sin Nombre orthohantavirus
-0.4777309	0.01700646	0.30011225	Human astrovirus-1
-0.1263379	0.02003978	0.31306129	Molluscum contagiosum virus
0.17395464	0.02010134	0.31306129	Tai Forest ebolavirus
0.20006486	0.02055925	0.31306129	Human papillomavirus type 40
-0.2605805	0.02354206	0.34289524	Simian virus 12
0.16021833	0.02744398	0.37163481	Human papillomavirus type 13
-0.5008972	0.02956515	0.37163481	Human parainfluenza 3 virus
-0.4573757	0.03330843	0.37163481	Human astrovirus-8
-1.7875789	0.03352913	0.37163481	Human respiratory syncytial virus
-1.7054823	0.03490607	0.37163481	Rhinovirus B
0.11561456	0.03535585	0.37163481	Human papillomavirus type 19
-0.5194499	0.03543062	0.37163481	Human parainfluenza 4a virus
-0.9443496	0.03605986	0.37163481	Human herpesvirus 3
0.11813685	0.03747745	0.37163481	Human coronavirus EMC
-0.1049473	0.03918074	0.37163481	Camelpox virus
-1.3751532	0.03953773	0.37163481	Hepatitis C virus genotype 1a
0.15286748	0.03955598	0.37163481	Cercopithecine alphaherpesvirus 2
-0.2008581	0.03993688	0.37163481	KI polyomavirus
-0.9028937	0.04143311	0.37513762	Human respiratory syncytial virus A
-1.2423726	0.04327934	0.38154156	Hepatitis C virus genotype 1c
-0.3173811	0.04500343	0.38656795	WU polyomavirus
0.13798471	0.04632022	0.38793182	Human papillomavirus type 61

Supplemental Table 3. Reactivity of donor human autoantibody targets by donor HIV status (extension of Figure 3)

Note: Only peptides of autoantibodies with raw p value<0.05 were included in this table. Log2 fold change represents the log2 ratio of reactivity of a peptide between donors with HIV vs donors without HIV, and a positive log2 fold change suggests reactivity of the autoantibody peptide was positively associated with donor HIV status.

log2 fold change	p value	q value	Autoantibody
-1.5139732	0.00071662	0.67495955	capping protein, Arp2/3 and myosin-I linker protein 3
3.40419355	0.00123169	0.67495955	uncharacterized protein C12orf40 isoform 1
-7.3149528	0.00259967	0.67495955	stathmin-4 isoform 1
33.5795161	0.00284443	0.67495955	hypothetical protein XP
2.9643391	0.00397105	0.67495955	splicing factor, suppressor of white-apricot homolog isoform 2
-2.2908694	0.00441811	0.67495955	capping protein, Arp2/3 and myosin-I linker protein 3
-1.9147482	0.00456273	0.67495955	protein piccolo isoform 1
29.0169355	0.0059823	0.67495955	hypothetical protein XP
1.62935484	0.00754551	0.67495955	C2 calcium-dependent domain-containing protein 4A
-1.2035012	0.00846838	0.67495955	cingulin
2.5583871	0.00937928	0.67495955	coiled-coil domain-containing protein 134 isoform 1 precursor
-3.6241778	0.00971378	0.67495955	protein phosphatase 1 regulatory subunit 12B isoform a
2.57322581	0.00980266	0.67495955	hypothetical protein XP
-0.9037018	0.01116444	0.67495955	bridging integrator 2 isoform 1
3.98564516	0.01143794	0.67495955	keratin, type II cytoskeletal 6C
15.1585484	0.01208442	0.67495955	hypothetical protein XP
17.5095161	0.01232064	0.67495955	hypothetical protein XP
3.0677262	0.01339676	0.67495955	synemin isoform A
-1.1424508	0.01472331	0.67495955	rho GTPase-activating protein 35
-1.0940716	0.0148897	0.67495955	C2 domain-containing protein 3 isoform 2
17.8129032	0.01542065	0.67495955	hypothetical protein XP
1.30322581	0.01597753	0.67495955	serine/threonine-protein phosphatase 1 regulatory subunit 10
-0.7408222	0.01683501	0.67495955	G-protein coupled receptor-associated sorting protein 1
1.89983871	0.01714428	0.67495955	ephrin type-A receptor 6 isoform a
-4.6149567	0.01835394	0.67495955	SPARC-related modular calcium-binding protein 1 isoform 1 precursor
-1.2628639	0.01851159	0.67495955	G-protein coupled receptor-associated sorting protein 2
-2.7914988	0.01861451	0.67495955	SNF-related serine/threonine-protein kinase isoform 1
-1.2324587	0.01874733	0.67495955	transcriptional activator GLI3
-0.7696617	0.01882272	0.67495955	cTAGE family member 2
-1.307443	0.01947424	0.67495955	histone-lysine N-methyltransferase 2C
9.89935484	0.01973237	0.67495955	hypothetical protein XP
-1.2076711	0.01982151	0.67495955	proliferation marker protein Ki-67 isoform 1
17.5081196	0.02147627	0.67495955	hypothetical protein XP
1.83974823	0.02161007	0.67495955	bcl-2-binding component 3 isoform 1
11.2940323	0.02164618	0.67495955	hypothetical protein XP
4.3245712	0.0218225	0.67495955	ankyrin-2 isoform 1
-3.3261959	0.02345873	0.67495955	SUN domain-containing protein 1 isoform a
1.38177419	0.02376302	0.67495955	protein phosphatase 1 regulatory subunit 12B isoform a
-4.2619276	0.02404163	0.67495955	SUN domain-containing protein 1 isoform a
2.87478757	0.02488957	0.67495955	ankyrin-2 isoform 1
2.58606216	0.02540235	0.67495955	voltage-dependent P/Q-type calcium channel subunit alpha-1A isoform 2
1.8583871	0.0257818	0.67495955	keratin, type II cytoskeletal 6B
2.02709677	0.02632686	0.67495955	keratin, type II cytoskeletal 6C
-3.416247	0.02824296	0.67495955	filaggrin-2
-0.8777616	0.02857903	0.67495955	ras and Rab interactor 3 isoform 1
-2.385295	0.02869312	0.67495955	ras-related GTP-binding protein C isoform 1
1.90053108	0.02924613	0.67495955	hypothetical protein XP
1.61354839	0.03005415	0.67495955	adiponectin receptor protein 1
2.30435484	0.03182302	0.67495955	rho guanine nucleotide exchange factor 40 isoform 1
0.63629032	0.03385021	0.67495955	protein Wnt-9b isoform 1 precursor
1.27992132	0.03385138	0.67495955	FH1/FH2 domain-containing protein 3 isoform 1
-2.1277026	0.0353138	0.67495955	SAM and SH3 domain-containing protein 1 isoform 1

-2.1590323	0.03544635	0.67495955	hypothetical protein
9.7216129	0.03550638	0.67495955	hypothetical protein XP
-3.1534264	0.03581698	0.67495955	SNF-related serine/threonine-protein kinase isoform 1
1.8029465	0.0371669	0.67495955	NUT family member 2D
-1.256668	0.03823256	0.67495955	protein JBTS17
-1.5245004	0.03933363	0.67495955	calcium-dependent secretion activator 1 isoform 1
1.04580645	0.03965024	0.67495955	synemin isoform A
0.99677419	0.03972446	0.67495955	kanadaptin
2.87761212	0.04018902	0.67495955	zinc finger protein 92 isoform 1
-1.8578442	0.04023294	0.67495955	zinc finger X-chromosomal protein isoform 3
-1.6102518	0.04098197	0.67495955	AT-rich interactive domain-containing protein 5B isoform 1
1.89741935	0.04148738	0.67495955	cullin-associated NEDD8-dissociated protein 1 isoform 1
2.63693548	0.04167541	0.67495955	eukaryotic translation initiation factor 4 gamma 1 isoform 1
-1.0737097	0.04181102	0.67495955	pleckstrin homology domain-containing family H member 3 precursor
1.03537766	0.04235683	0.67495955	AF4/FMR2 family member 3 isoform 2
0.82903226	0.04306254	0.67495955	centrosomal protein of 290 kDa
1.44290716	0.0438416	0.67495955	ER degradation-enhancing alpha-mannosidase-like protein 1
1.91599135	0.04387535	0.67495955	dynein heavy chain 9, axonemal isoform 2
-0.6414398	0.04394258	0.67495955	coiled-coil domain-containing protein 33 isoform 2
3.84612903	0.04480729	0.67495955	synaptotagmin-like protein 2 isoform c
1.5316129	0.04506111	0.67495955	arf-GAP with GTPase, ANK repeat and PH domain-containing protein 3 isoform a
-1.1128521	0.04515593	0.67495955	tyrosyl-DNA phosphodiesterase 1 isoform a
0.82903226	0.04528834	0.67495955	voltage-dependent T-type calcium channel subunit alpha-1I isoform a
1.05870968	0.04647212	0.67495955	centrosomal protein of 78 kDa isoform a
3.22564516	0.04668029	0.67495955	synaptotagmin-like protein 2 isoform c
1.12244689	0.04701223	0.67495955	aprataxin isoform a
1.60822581	0.04737876	0.67495955	structural maintenance of chromosomes flexible hinge domain-containing protein 1
-1.4911015	0.04754166	0.67495955	endonuclease domain-containing 1 protein precursor
-1.9037018	0.04781143	0.67495955	leucine zipper protein 1
0.91580645	0.04785667	0.67495955	protein eva-1 homolog C isoform 1 precursor
12.5603816	0.04895073	0.67495955	hypothetical protein XP
1.39226987	0.04895302	0.67495955	ribosomal protein S6 kinase beta-2
1.12370968	0.04957242	0.67495955	putative uncharacterized protein C20orf78 isoform 1
1.74244689	0.04971065	0.67495955	zinc finger protein 486

Supplemental Table 4. Reactivity of donor viral antibody targets and allograft rejection among recipients (extension of Figure 5A)

Note: Only viral species with raw p value<0.05 were included in this table. Log2 fold change represents the log2 ratio of a viral species between rejection vs no rejection, and a positive log2 fold change suggests reactivity of the viral species was positively associated with rejection.

log2 fold change	p value	q value	Viral species
0.921977818	0.000034698	0.01210959	Human adenovirus A serotype 12
0.592284653	0.00079011	0.08838446	Human adenovirus A serotype 18
0.608653385	0.0009587	0.08838446	Human adenovirus A serotype 31
1.471358087	0.00106574	0.08838446	Human adenovirus 55
0.739882992	0.00142125	0.08838446	Human adenovirus F serotype 40
2.468602551	0.0015195	0.08838446	Human adenovirus C serotype 2
0.536627374	0.00323715	0.14487367	Human adenovirus F serotype 41
0.863699002	0.00332089	0.14487367	Human adenovirus E serotype 4
0.2097705	0.00401277	0.14965096	Mumps virus
1.444719808	0.00428799	0.14965096	Human adenovirus B serotype 16
0.523125481	0.004727	0.14997476	Human metapneumovirus
0.727376515	0.02106267	0.61257263	Human adenovirus C serotype 5
0.728467968	0.03043182	0.80919258	Human adenovirus 14
0.127177282	0.03707767	0.80919258	Bundibugyo ebolavirus
0.193270379	0.03753961	0.80919258	Sindbis virus subtype Ockelbo
0.178113013	0.03812415	0.80919258	Simian foamy virus type 1

Supplemental Table 5. Reactivity of donor human autoantibody targets and acute rejections among recipients (extension of Figure 6)

Note: Only peptides of autoantibodies with raw p value<0.05 were included in this table. Log2 fold change represents the log2 ratio of reactivity of a autoantibody peptide between rejection vs no rejection, and a positive log2 fold change suggests reactivity of the autoantibody peptide was positively associated with rejection.

log2 fold change	p value	q value	Peptides of autoantibodies
3.74123418	0.0021464	0.91373216	hypothetical protein LOC390760
1.73569093	0.00402246	0.91373216	zinc finger protein 676
1.43165084	0.00421624	0.91373216	insulin-like peptide INSL6 precursor
3.53337025	0.00937175	0.91373216	ankyrin repeat domain-containing protein 50 isoform 1
1.4975	0.00946652	0.91373216	uncharacterized protein KIAA1109
1.43281646	0.01001021	0.91373216	SH3 and multiple ankyrin repeat domains protein 1
2.4239557	0.01014586	0.91373216	potassium/sodium hyperpolarization-activated cyclic nucleotide-gated channel 4
5.05144515	0.01235376	0.91373216	transcription factor SPT20 homolog-like 1 precursor
3.36243671	0.01391785	0.91373216	histone acetyltransferase KAT7 isoform 1
2.7028692	0.01611943	0.91373216	histone acetyltransferase KAT7 isoform 1
1.47920359	0.01695036	0.91373216	oxygen-regulated protein 1
1.1555116	0.02005268	0.91373216	bromo adjacent homology domain-containing 1 protein isoform a
1.96714135	0.02151399	0.91373216	PHD finger protein 19 isoform a
2.81012658	0.02371667	0.91373216	rap1 GTPase-activating protein 1 isoform b
5.61739979	0.02386964	0.91373216	F-box-like/WD repeat-containing protein TBL1XR1 isoform 1
1.28522679	0.02440402	0.91373216	transcription termination factor 1 isoform 1
0.96380274	0.02627251	0.91373216	zinc finger protein 676
3.8958808	0.02844267	0.91373216	hypothetical protein XP
1.87044304	0.02879495	0.91373216	calcium-dependent secretion activator 1 isoform 1
5.06482068	0.02888766	0.91373216	zinc finger protein with KRAB and SCAN domains 2
1.74945675	0.03165535	0.91373216	protein phosphatase 1 regulatory subunit 12B isoform a
1.42945675	0.03736571	0.91373216	EKC/KEOPS complex subunit GON7
4.14253165	0.03855123	0.91373216	voltage-dependent R-type calcium channel subunit alpha-1E isoform 3
2.048423	0.03910064	0.91373216	A disintegrin and metalloproteinase with thrombospondin motifs 3 preproprotein
1.60609705	0.04302606	0.91373216	nuclear receptor subfamily 1 group I member 2 isoform 2
-3.6744093	0.04671272	0.91373216	activin receptor type-2B precursor
-2.7113924	0.04920173	0.91373216	glutaredoxin-3 isoform 1
2.38957806	0.04928123	0.91373216	arf-GAP with GTPase, ANK repeat and PH domain-containing protein 2 isoform PIKE-L
